# The quantification of the psychiatric revolution: a quasi-natural experiment of the suicide impact of the Basaglia Law

**DOI:** 10.1093/eurpub/ckaa011

**Published:** 2020-02-19

**Authors:** Caterina Ronchetti, Veronica Toffolutti, Martin McKee, David Stuckler

**Affiliations:** c1 Department of Social and Political Science, Bocconi University, Milano, Italy; c2 “Carlo F. Dondena” Centre for Research on Social Dynamics and Public Policies, Bocconi University, Milano, Italy; c3 Department of Public Health and Policy, London School of Hygiene and Tropical Medicine, London, UK

## Abstract

**Background:**

The Italian 180/1978 reform abolishing asylums is one of the most contested mental health programs ever implemented. It aimed to shift care of mental illness into the community improving outcomes and reducing expenditure. It was a model for successive deinstitutionalization initiatives across Europe and North America. However, there were longstanding concerns that, without expansion of community care, it may have deprived patients with mental illness access to support, placing them at increased risk of suicide.

**Methods:**

Regression discontinuity models were used to quantify the association between the number of suicides and the introduction of the Basaglia Law, disaggregated by age-group and gender, covering 20 Italian regions during the period 1975–84. Models were adjusted for potential socio-demographic confounding factors, region-specific fixed effects and pre-existing time-trends.

**Results:**

Italian regions implemented the Basaglia Law to varying degrees over time. We observed that, after adjusting for pre-existing time trends, the implementation was associated with a consistent increase in the number of suicides for all the age-groups [incidence rate ratio, age 15–44: 1.29, 95% confidence interval (CI) 1.18–1.41; age 45–74: 1.45, 95% CI 1.37–1.54] and for both genders (males: 1.47, 95% CI 1.41–1.53; females: 1.36, 95% CI 1.25–1.47). Hospital closure appeared to be an important mediating mechanism.

**Conclusions:**

The Basaglia Law was associated with a significant increase in the number of suicides, with evidence of an association with closures of facilities, leaving those with mental illness with nowhere to go, as the envisioned community care structures failed to be developed as originally planned.

## Introduction

‘*La follia è una condizione umana. In noi la follia esiste ed è presente come lo è la ragione. Il problema è che la società, per dirsi civile, dovrebbe accettare tanto la ragione quanto la follia, invece incarica una scienza, la psichiatria, di tradurre la follia in malattia allo scopo di eliminarla. Il manicomio ha qui la sua ragion d’essere.’*

*‘Madness is a human condition. Madness is present in everyone as it is reasoning. The problem is that society, to be called civil, should accept both reason and folly, rather than requiring a science, psychiatry, to translate madness into illness in order to eliminate it. Asylums have the goal to eliminate madness’.*

*Franco Basaglia, 1973*

One of the most radical, but contested, mental health reforms ever implemented in Europe was in Italy in 1978, when Law 180/1978 initiated the closure of Italian asylums. In his famous 1964 report entitled *The Destruction of the Mental Hospital as a Place of Institutionalisation*, Dr Franco Basaglia, the leading advocate of the reform whose name would be attached to the 1978 Law, argued that this would improve the mental health of those who had been institutionalized. In his own words, he sought to ‘transform the whole system from the edge, from the extreme periphery.’^1^

The core idea of the Basaglia Law was to close psychiatric hospitals and shift care to the new Community Mental Health Centers (CMHCs). Basaglia viewed asylums as ‘deposits where people believe the mad (*i pazzi*) are sent, where intellectuals believe the lunatics (*i folli*) are sent and where doctors believe mental patients are looked after and treated. For the mad, the lunatic and the mental patient it is a locked, oppressive and total institution where punitive, prison-like rules are applied, in order to slowly eliminate its own contents’.^2^ Under the new regime, from 1981 onwards, patients would no longer be admitted to psychiatric hospitals, and after 1982 hospitalization was an option only for those patients previously hospitalized. Psychiatric wards were still permitted in general hospitals, but the maximum number of beds was reduced to 15. The criteria for compulsory hospitalization became stricter and patients could be detained for a maximum of 7 days.^3^ The Law led to the creation of a new psychiatric care system managed by a newly created network of regional departments of mental health (Dipartamento di Salute Mentale—DSM) and corresponding Mental Health Centers (Centri di Salute Mentale—CSM), managed at regional level.^3^

From the outset, the Law generated controversy. First, it treated suicide as a primarily social issue, downplaying the importance of medical treatment. Second, Basaglia was blamed for ‘having abandoned patients to their fate’, as patients without accompanying community support were left with nowhere to go and deprived of access to care.^1^ Earlier, when he initiated a radically new approach to psychiatry in the asylum of the Italian city of Gorizia, he was twice charged with manslaughter after two of his patients were released and killed relatives.^1^ Thus, it has been hypothesized that, perversely, the Law may have had unintended consequences, including an increased risks of suicide.

To our knowledge, only a few studies have investigated the potential impacts of the Basaglia Law. This gap likely reflects a lack of data. One small-scale qualitative study tracked 163 people released from the Mombello mental hospital and reintegrated in the local communities in 1999, with follow up until 2002,^4^ finding no major change in mental health or functional capacity. However, it came over a decade after the Law was implemented, by which time community services are likely to have evolved and there was no comparison group. Another descriptive study evaluated the impact of the Law on incarceration rates, covering the period 1978–94.^5^ It reported that, pre-Basaglia, the vast majority of prison inmates had committed violent crimes (such as homicides), whereas afterwards they included very large numbers suffering from psychological distress*.* Yet given its observational design, and lack of statistical analysis, its conclusions were contested.^6–9^

Here, we plug these gaps in the literature in two important ways, taking advantage of the marked variation in the Law’s implementation, perhaps inevitable in a country with large regional disparities in wealth and administrative functioning.^10^ Central and Northern regions of Italy implemented the Law rapidly while those in the South virtually ignored it.

First, we constructed, for the first time to our knowledge, a quantitative dataset from archival information that captures this differential progress in implanting the Law across Italian regions and link it to data on the number of suicide by age and gender. Second, we use this dataset in a quasi-natural experiment to test the impact of the Basaglia Law on suicide, exploiting the regional variation.

## Methods

We collected suicide data from several sources. We extracted data on the number of suicides at regional level from historical archives of the Statistical Sanitary Yearbooks for the period 1975–84.^11^ This provided data at regional level for 19 region and 2 provinces (Trento and Bolzano). Consistent with many other studies, given their small size we combined the data from Trento and Bolzano to create an entity we term Trentino-South Tyrol.

Suicide is classified as self-injury in the tabulations of cause of death that we used. As a sudden and usually unexplained cause, each case is investigated by the authorities. Up to 1979, this definition included only ‘suicide’, after which it was extended to include ‘suicide and self-harm’. Given the timing, we wondered if this was due to a change in coding. However, the International Statistical Classification of Diseases (ICD) codes for suicide and self-inflicted injury remained the same (E950-E959) in ICD-8 (pre-1979) and ICD-9 (post-1979) and this seems to have been simply a change of labeling in the Italian publications. As we are interested in understanding the potential impact on the population of each region, we exclude all cases where the region of residence of the deceased is unknown. We use the total number of suicides in each region by the resident population, which were taken from the census data. The data are separated by year, region and gender, and disaggregated into two age bands, 15–44 and 45–74 years old, as reported in the original data. Supplementary tables A1a and A1b provide further details and descriptive statistics.

To gather evidence of the mediating role of psychiatric hospital closure, we compute the cumulative percentage of asylums closed during the period of interest: 1977–84. The data come from the Italian Ministry of Cultural Heritage and Activities archive.^11^

### Statistical models

The association between suicides rates and implementation of the Basaglia Law at regional level is analyzed using the following equation:

$$\;{\log(E(S}_{it}|{\mathrm{Basaglia}}_{it}))\;=\;\beta {\cdot \mathrm{Basaglia}}_{it}+\gamma {\cdot \mathrm{Region}}_{i}$$

where *i* is the region and age-group or gender, *t* is the year, *S* is the annual number of suicides, Basaglia is a dummy variable equal to 1 after the Law’s implementation (after 1978) and 0 otherwise. Region represents regional fixed-effects. The implementation of the Law was the same across the entire country so the dummy Basaglia will take a value equal to 1 after 1978 in all regions, whereas the regional fixed effect should control for differentials in the numbers of suicides at regional level. Here, we use a Poisson regression model and we report the estimation results in terms of incidence rate ratios.

The coefficient of interest in this model is *β*, whose complement to 1 represents the percentage change in suicides associated with the implementation of the Basaglia Law. We used robust standard errors to reflect non-independent sampling.

To further explore any possible association between implementation of the Basaglia Law and a change in the number of suicides, we present the estimation results by using the percentage of asylums closed in each region and year as a main explanatory variable, as described in Equation 2.

$$\;{\log(E(S}_{it}|{\mathrm{Closure}}_{it}))\;=\;\vartheta {\cdot \mathrm{Closure}}_{it}+\gamma {\cdot \mathrm{Region}}_{i}+\tau \;{\cdot \;\mathrm{Time}}_{t}$$

where Closure represents the cumulative percentage of asylums closed. Time represents a time trend. The coefficient of interest in this model is *ϑ*, whose complement to 1 represents the percentage change in the number of suicides associated with a percentage point increase in the psychiatric hospitals closed. Again, we used robust standard errors. All models were estimated using STATA version 15.

However, in nine regions no asylums were closed in the period under examination. According to the Italian Ministry of Cultural Heritage and Activities, Tuscany and Lazio closed all their psychiatric hospitals in 1978, immediately after the introduction of the Law, while Liguria, Trentino-South Tyrol and Apulia did so after 1984, beyond our observational period. Abruzzo and Sicily closed some asylums in 1978 and the remainder after 1984. Finally, Aosta-Valley and Molise did not host any asylums: mentally ill patients were sent to Piedmont and to Campania, respectively. We would therefore not expect any significant change in them so, as a robustness check, we undertook a separate analysis restricted to those regions. The results are shown in the table 3.

**Table 3:** Change in the number of suicides associated with the implementation of the Basaglia Law—placebo test on the nine regions which did not show any variation in the number of asylums closed—Poisson model

	Ages 15–44	Ages 45–74	Males	Females
Implementation of Basaglia Law	0.87	0.96	1.06	1.09
95% CI	[0.45–1.65]	[0.84–1.14]	[0.94–1.19]	[0.94–1.25]
Regional dummies	Yes	Yes	Yes	Yes
Year dummies	Yes	Yes	Yes	Yes

*Source*: Data from ISTAT—Statistical Sanitary Yearbooks (1975–1984) and ISTAT—Italian Statistical Yearbooks (1976–1985). Number of regions = 9, number of region-years = 90. 95% confidence intervals in brackets. The dependent variable represents the number of suicides. This has been regressed through an Poisson model on a dummy variable equal to 1 after 1978 (implementation of Basaglia Law), regional fixed-effect, year linear trend.

## Results

**Figure 1:**
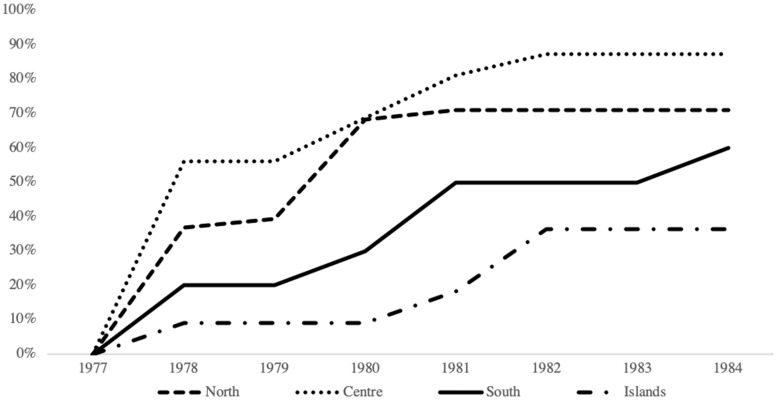
Time-trend in the cumulative percentage of asylums closures, by region *Source:* Author’s calculations using data from the Italian Ministry of Cultural Heritage and Activities

Figure 1 presents the time-trend in psychiatric hospital closures associated with the Basaglia Law. Given the large number of regions, the data are aggregated for presentational purposes into four macro-areas: North (Aosta-Valley, Lombardy, Trentino-South Tyrol, Veneto, Friuli-Venezia Giulia, Liguria and Emilia Romagna), Centre (Tuscany, Umbria, Marches and Lazio), South (Abruzzo, Molise, Campania, Apulia, Basilicata and Calabria) and Islands (Sardinia and Sicily) and represent rates per 100 000 inhabitants.

The difference in closure rates across the country is starkly apparent. The Northern regions changed most rapidly, whereas the Central and the Southern regions did so at a much slower rate. However, Tuscany, in the central part of the country, was the region most compliant and the only one to close all asylums after enactment of the Basaglia Law. Further details on changes in each region are presented in Supplementary table A1. Next, we proceed to identify any association between differential implementation of the Law and suicides across regions.

Table 1 presents the results of our statistical models disaggregated by age (15–44 vis-à-vis 45–74), in the first two columns, and gender in the last two columns. We observed an increase in the number of suicides associated with the introduction of the Basaglia Law in all age groups. This was 29% in the younger group [1.29, 95% confidence interval (CI) 1.18–1.41] and 45% in the older one (1.45, 95% CI 1.37–1.54). The results are consistent across genders, with the point estimate slightly higher in men (men: 1.47, 95% CI 1.41–1.53, women 1.36, 95% CI 1.25–1.47).

**Table 1:** Change in the number of suicides associated with the Basaglia Law implementation, results separately by age-group and gender—Poisson model

	Ages 15–44	Ages 45–74	Males	Females
Implementation of Basaglia Law	1.29	1.45	1.47	1.36
95% CI	[1.18–1.41]	[1.37–1.54]	[1.41–1.53]	[1.25–1.47]
Regional dummies	Yes	Yes	Yes	Yes
Year dummies	Yes	Yes	Yes	Yes

*Note:* Coefficients represents Incidence Rate Ratios.

*Source:* Data from ISTAT—Statistical Sanitary Yearbooks (1975–1984) and ISTAT—Italian Statistical Yearbooks (1976–1985). Number of regions = 20, number of region-years = 200. 95% confidence intervals calculated with Robust Standard Errors are presented in brackets. The dependent variable represents the number of suicides. This has been regressed through an Poisson model on a dummy variable equal to 1 after 1978 (implementation of Basaglia Law), regional fixed-effect, year linear trend.

Table 2 presents the estimation results using the potential mediating role of psychiatric hospital closure. The first two columns present the results by age-group (15–44 vis-à-vis 45–74), whereas the last two columns present the results separately by gender. Asylum closure appears to be significantly associated with an increase in the number of suicides for males only by about 8% (1.08, 95% CI 1.01–1.16) but in the youngest age group (both sexes) by about 31% (1.31, 95% CI 1.04–1.65). The figures for older people and women do not reach significance.

**Table 2:** Change in the number of suicides associated with the percentage of asylums closed

	Ages 15–44	Ages 45–74	Males	Females
Closure	1.31	0.99	1.08	0.97
95% CI	[1.04–1.65]	[0.88–1.11]	[1.01–1.15]	[0.85–1.11]
Regional dummies	Yes	Yes	Yes	Yes
Year dummies	Yes	Yes	Yes	Yes

*Note:* Coefficients represents Incidence Rate Ratios.

*Source*: Data from ISTAT—Statistical Sanitary Yearbooks (1975–1984) and ISTAT—Italian Statistical Yearbooks (1976–1985). Number of regions = 20, number of region-years = 200. 95% confidence intervals calculated with Robust Standard Errors are presented in brackets. The dependent variable represents the number of suicides. This has been regressed through an Poisson model on the cumulative percentage of psychiatric hospitals closed at regional level equal to 1 after 1978 (implementation of Basaglia Law), regional fixed-effect, year linear trend.

### Robustness checks

Nine of the regions experienced no closures of asylums. We would therefore not expect any significant change in them so, as a robustness check, we undertook a separate analysis restricted to those regions. The results are shown in table 3 and support our interpretation in that there was no significant effect in regions experiencing no closures.

To gather further evidence on the possible impact of the Laws implementation on the number of suicide rates, we test, in Supplementary table A2 (a. separately by age-groups and b. separately by gender) sensitivity to variation in implementation. More specifically, we recode the dummy Basaglia equal to 1 from 1981 onwards and 0 otherwise. We chose 1981 because admission of new patients into psychiatric hospitals was prevented from that year onwards. The results are consistent with the main findings, although of smaller magnitude. Our results show that, after 1981, suicide rates increased by about 12% (1.12, 95% CI 1.04–1.21) for individuals aged 45–74 years old. In contrast to our main results, our evidence shows that after 1981, there was no increase in the suicide rate for individuals aged below 44 years. When we look at gender differences, we find that, starting in 1981, Italy recorded an increase in suicide mortality of about 15% for males (1.15 95% CI 1.09–1.23) of about 4% for females (1.04, 95% CI 1.01–1.19).

## Discussion

‘There is no health without mental health. For citizens, mental health is a resource, which enables them to realize their intellectual and emotional potential and to find and fulfill their roles in social, school and working life. For societies, good mental health of citizens contributes to prosperity, solidarity and social justice. In contrast, mental ill health imposes manifold costs, losses and burdens on citizens and societal systems’. Those are the words used by the European Commission in the Green Paper establishing an EU-strategy on mental health.^12^ This sentence highlights not only the commitment of the EU on mental health issues, but also their social relevance. Currently, we take for granted that the dignity of people with mental illness should be safeguarded, but this was not always the case. It was Basaglia, along with a few other pioneers, who challenged the inhumanity that characterized much psychiatric care and ‘allowed Italy to be the first country in the world to abolish psychiatric hospitals, paving the way for similar processes to unravel around the world’.^13^

Even now, the Law is one of the most contested mental health programs across Europe. Many have tried to investigate its potential effects either using qualitative or quantitative methods. However, the evidence is far from clear-cut.

In this article, we investigated the association between the implementation of the Law and suicide rates across Italy, a controversial topic already widely discussed before 1978. Indeed, at the time one of the main arguments against its implementation was the potential for a rise in suicide rates, as the closure of Asylums would have left mentally ill people with nowhere to go. However, supporters of the Law argued that suicides did not increase after the implementation of Law 180/1978 and that Italy had among the lowest suicide rates in Europe, a continent in which many countries even now operate large mental hospitals.^14^

Our results show that there was an increase in the suicide rates associated with the implementation of the Law. The results, however, offer a more nuanced interpretation of who was affected. We found that it was among all age-groups, with the middle-aged people (aged between 45 and 174 year) (age 14–44: 1.29, 95% CI 1.18–1.41; age 45–74: 1.45, 95% CI 1.37–1.54) and both genders showing an increase by about 40% (men: 1.47, 95% CI 1.41–1.53, women 1.36, 95% CI 1.25–1.47).

The study has several limitations. First, and most obviously, it is an ecological analysis. We cannot know whether those who kill themselves have been in asylums or, if they remained, would have been admitted. While many suicides are among those who have come to medical attention, many others are not,^15^^,^^16^ although among those that are in contact with health services, suicide ideation, if looked for, does have some predictive power.^17^ Second, we cannot exclude the possibility that other changes, for example in the economy, may have played a role, although it is difficult to envisage what these might be at the regional level that would have coincided with the different times when the Law was implemented. Third, our analysis is limited to a short time period. Further studies are needed in to assess whether the situation changed in later years and whether there was any lag in the reform’s effects, although given the natural history of suicide and its precedents, this is unlikely. Fourth, we have no data on the creation and quality of new services such as CMHCs^18^^,^^19^ which, if functioning well, would be expected to mitigate adverse effects of deinstitutionalization. Fifth, there are concerns about the validity of suicide data in all countries, many studies, including in an international comparison explored the possibility that other diagnostic codes might be substituted for suicide. This may be less so in Italy than elsewhere as it was one of the countries that met the quality threshold.^20^

Further research is needed to understand the wider consequences of the implementation of the Basaglia Law, beyond suicides rates. In particular, it will be useful to know whether its financial goals were achieved—did the reform increase or decrease government spending, was homelessness reduced, and how did the prison population change, and in particular have prisons taken over the role once fulfilled by asylums. Finally, there remains a major question for researchers as to whether mental health in Italy has significantly improved since 1978.

Our results allow us to speculate on two possible mechanisms underlying what we find. First, the quality of mental health care might have declined after the introduction of the Law. Burti (2001) reports that the quality of mental-care, after the reform, was poor and declining and only a handful of programs offering rehabilitation in the community were available.^21^ Besides, a 1984 nationwide survey undertaken by CENSIS, showed that both in and out-patient facilities were available to more than 80% of the population in their own catchment areas. Concomitantly, 236 additional hospitals (amounting to 3113 additional hospital beds) were opened. Unfortunately, this amounted only to 60% of the originally planned ones. Other residential facilities, with a total number of 2901 beds were open at the same time, but the number of admissions to public hospitals halved before the reform from 60 000 to 30 000.^22^

Second, even if conditions had improved by the 1970s, Basaglia’s description of conditions in the asylums make clear that they were terrible places to be, with patients often naked, deeply disturbed, and lying in excrement. The argument against them was overwhelming. But closing them was only one part of any solution. It left mentally ill people with nowhere to go, as the CMHCs were not created as originally planned in the Law. Therefore, even if its goal was laudable, the Law’s implementation lacked one fundamental element, the creation of a safe place where mentally ill people could live. The alternatives, such as loneliness, isolation, and for some, prison, are entirely plausible risk factors for suicide.

## Supplementary data

Supplementary data are available at *EURPUB* online.

## Funding

V.T. and D.S. are supported by an ERC [313590-HRES]. V.T. is also supported by ERC [694145- IFAMID]. D.S. is also funded by Wellcome Trust.

*Conflicts of interest*: All authors have completed the ICMJE uniform disclosure form. The authors have no financial relationships with any organizations that might have an interest in the submitted work in the previous 5 years, no other relationships or activities that could appear to have influenced the submitted work.

**Key points**

- The Italian 180/1978 reform abolishing asylums is one of the most contested mental health programs ever implemented.
- Regression discontinuity models were used to quantify the association between suicide rates and the introduction of the Basaglia Law.
- The Basaglia Law was associated with a significant increase in suicide rates.

## Supplementary Material

ckaa011_Supplementary_Data
