# Supplementary material for: The quantification of the psychiatric revolution: a quasi-natural experiment of the suicide impact of the Basaglia Law
Source: Eur J Public Health. 2020 Feb 19;30(3):492–6. doi: 10.1093/eurpub/ckaa011 (PMC7292351; doi:10.1093/eurpub/ckaa011)
Supplement: ckaa011_Supplementary_Data [file ckaa011_supplementary_data.docx]

**Web Appendix 1:**

**Table A1:** Descriptive statistics

**Table A2:** Change in the percentage of suicides associated with the available hospital beds.

**Table A1:** Descriptive statistics

1. Time trend in the number of suicide at regional level

|  | 1975 | 1976 | 1977 | 1978 | 1979 | 1980 | 1981 | 1982 | 1983 | 1984 |
| --- | --- | --- | --- | --- | --- | --- | --- | --- | --- | --- |
| North | 1395 | 1329 | 1979 | 2094 | 2267 | 2263 | 2228 | 2358 | 2445 | 2487 |
| Centre | 536 | 601 | 707 | 666 | 779 | 814 | 706 | 768 | 810 | 854 |
| South | 480 | 442 | 515 | 501 | 524 | 647 | 549 | 626 | 620 | 653 |
| Islands | 306 | 329 | 297 | 377 | 370 | 380 | 426 | 415 | 395 | 431 |

*Source:* Author’s calculations using data from ISTAT – Statistical Sanitary Yearbooks (1975-1984) and ISTAT – Italian Statistical Yearbooks (1976-1985).

1. Time-trend in Asylum closure (cumulative percentage) at regional level

|  | 1978 | 1979 | 1980 | 1981 | 1982 | 1983 | 1984 |
| --- | --- | --- | --- | --- | --- | --- | --- |
| Piedmont | 80% | 80% | 80% | 100% | 100% | 100% | 100% |
| Aosta-Valley | 0% | 0% | 0% | 0% | 0% | 0% | 0% |
| Liguria | 0% | 0% | 0% | 0% | 0% | 0% | 0% |
| Lombardy | 29% | 29% | 43% | 43% | 43% | 43% | 43% |
| Trentino-South Tyrol | 0% | 0% | 0% | 0% | 0% | 0% | 0% |
| Veneto | 36% | 43% | 86% | 86% | 86% | 86% | 86% |
| Friuli-Venezia Giulia | 67% | 67% | 100% | 100% | 100% | 100% | 100% |
| Emilia-Romagna | 20% | 20% | 80% | 80% | 80% | 80% | 80% |
| Tuscany | 100% | 100% | 100% | 100% | 100% | 100% | 100% |
| Umbria | 0% | 0% | 100% | 100% | 100% | 100% | 100% |
| Marches | 17% | 17% | 33% | 67% | 83% | 83% | 83% |
| Lazio | 100% | 100% | 100% | 100% | 100% | 100% | 100% |
| Abruzzo | 50% | 50% | 50% | 50% | 50% | 50% | 50% |
| Molise | 0% | 0% | 0% | 0% | 0% | 0% | 0% |
| Campania | 0% | 0% | 33% | 67% | 67% | 67% | 67% |
| Apulia | 0% | 0% | 0% | 0% | 0% | 0% | 0% |
| Basilicata | 0% | 0% | 0% | 100% | 100% | 100% | 100% |
| Calabria | 50% | 50% | 50% | 50% | 50% | 50% | 100% |
| Sicily | 13% | 13% | 13% | 13% | 13% | 13% | 13% |
| Sardinia | 0% | 0% | 0% | 33% | 100% | 100% | 100% |

*Source*: Author’s calculations using data from the Italian Ministry of Cultural Heritage and Activities.

**Table A2:** Change in the number of suicides associated with the Basaglia Law implementation, Poisson Model

1. Results separately by age-group.

|  | Ages 15-44 | Ages 45-74 |
| --- | --- | --- |
| Implementation of  Basaglia Law | 1.05 | 1.12 |
| 95% CI | [0.98, 1.12] | [1.04, 1.21] |
| Regional dummies | Yes | Yes |
| Year dummies | Yes | Yes |

1. Results separately by gender

|  | Males | Females |
| --- | --- | --- |
| Implementation of  Basaglia Law | 1.15 | 1.04 |
| 95% CI | [1.09, 1.23] | [1.01, 1.19] |
| Regional dummies | Yes | Yes |
| Year dummies | Yes | Yes |

*Notes:* * *p* < 0.05 ** *p* < 0.01 *** *p* < 0.001. Source: Data from ISTAT – Statistical Sanitary Yearbooks (1975-1984) and ISTAT – Italian Statistical Yearbooks (1976-1985). Number of regions = 20, number of region-years = 200. Robust standard errors in parenthesis.

The dependent variable represents the number of suicides. This has been regressed using an Poisson model on a dummy variable equal to 1 after 1981 (Implementation of Basaglia Law) , regional fixed-effect, year linear trend.
